# COVID‐19 presenting as severe acute hepatitis in a pediatric patient with thalassemia minor: A case report

**DOI:** 10.1002/ccr3.8955

**Published:** 2024-05-24

**Authors:** Syed Abdan Jamalvi, Sameer Abdul Rauf, Atika Sherali, Syed Khizar Ali, Hussain Haider Shah, Filza Jamalvi, Fnu Yogeeta, Tirth Dave

**Affiliations:** ^1^ Department of Internal Medicine Liaquat National Medical College Karachi Pakistan; ^2^ Department of Pediatrics Liaquat National Medical College Karachi Pakistan; ^3^ Bukovinian State Medical University Chernivtsi Ukraine

**Keywords:** coronavirus, COVID‐19, hepatitis, SARS‐CoV‐2, thalassemia

## Abstract

**Key Clinical Message:**

This case emphasizes the significance of COVID‐19 in pediatric patients presenting with unusual hepatic manifestations, urging clinicians to broaden their diagnostic lens. The unexpected elevation of SARS‐CoV‐2 antibodies and the effective use of N‐acetyl cysteine highlight the importance of adaptability in treatment strategies.

**Abstract:**

This case report presents a unique manifestation of severe hepatic involvement in a 4‐year‐old girl with thalassemia minor and COVID‐19. Despite the absence of prominent respiratory symptoms, the patient exhibited jaundice, elevated liver enzymes, and coagulopathy. Initial suspicion of viral hepatitis was replaced by the discovery of significantly elevated SARS‐CoV‐2 antibodies. A multidisciplinary approach, including gastroenterology consultation and an extensive workup, was pivotal in ruling out alternative etiologies. Unconventional use of N‐acetyl cysteine contributed to clinical improvement, highlighting the need for adaptable treatment strategies. This case underscores the importance of heightened awareness in recognizing atypical presentations of COVID‐19 in pediatric patients, especially those with underlying health conditions. Further exploration into nuanced manifestations and treatment approaches is warranted for comprehensive clinical management.

## 1 INTRODUCTION

The global impact of the COVID‐19 pandemic has resulted in over 771,820,937 confirmed cases and more than 6,978,175 deaths as of November 2023 since its origin back in December 2019.^1^ Children account for 17.9% of the cumulative cases.^2^ The clinical symptoms may differ based on age and existing health conditions, but commonly, individuals experience respiratory manifestations like fever, cough, and general discomfort. Less prevalent symptoms include a sore throat, chest pain, chills, nausea, and vomiting.^3^

Hepatic manifestations of COVID‐19 noted a derangement of total bilirubin, aspartate aminotransferase (AST), alanine aminotransferase (ALT) and albumin levels.^4^ The exact mechanism underlying liver injury in the context of COVID‐19 infection remains not fully elucidated. However, several hypotheses have been suggested, encompassing the direct impact of the virus on cells, immune system dysfunction, systemic inflammatory response syndrome, cytokine storm, sepsis, vascular clotting, hypoxia, and ischemia–reperfusion injury.^5^, ^6^

We present a case of a 4‐year‐old girl with thalassemia minor who presented with fever, jaundice, and elevated liver enzymes. Despite initial suspicion of acute viral hepatitis and treatment initiation, further investigations revealed significantly elevated SARS‐CoV‐2 antibodies, highlighting a rare presentation of COVID‐19‐associated liver injury.

## 2 CASE HISTORY/ EXAMINATION

A 4‐year‐old girl presented to the emergency department with a complaint of fever and yellow discoloration of skin and sclera for the last 1 week, which was increasing gradually associated with dark yellow colored urine. She is a known case of beta thalassemia minor, with no ongoing treatment for it. On examination, the 4‐year‐old girl had normal growth parameters. She was well oriented, with the following vitals: temperature 37.4°C, blood pressure 79/63 mmHg, heart rate 110 beats/min, and 98% oxygen saturation at room air. She had jaundice of skin and sclera with no anemia, petechiae, bruises, enlarged lymph nodes and no peripheral stigmata of chronic liver disease. On abdominal examination, the abdomen was soft, not distended. There was epigastric tenderness, but no hepatosplenomegaly. Other systemic examination was unremarkable. (Table 1).

**TABLE 1:** shows the Laboratory results on admission.

Test	Result	Normal Range
Red blood cell count (RBC)	4.4 million/mcL	4.5–5.5 million/mcL
Hemoglobin (Hb)	11 g/dL	13.5–17.5 g/dL
Alkaline phosphatase (ALP)	277 U/L	30–120 U/L
Aspartate aminotransferase (AST)	1368 U/L	10–40 U/L
Alanine aminotransferase (ALT)	1247 U/L	7–56 U/L
Total bilirubin	17.12 mg/dL	0.3–1.0 mg/dL
Direct bilirubin	16.05 mg/dL	0.1–0.3 mg/dL
International normalized ratio (INR)	1.9	0.8–1.2
White blood cell count (WBC)	13.20 K/μl	4.5–11.0 K/μL
Platelet count	367 K/μl	150–400 K/μL
C‐reactive protein (CRP)	0.8 mg/L	0–10 mg/L

## 3 METHODS (DIFFERENTIAL DIAGNOSIS, INVESTIGATIONS, AND TREATMENT)

Based on history, examination, and initial lab findings, a diagnosis of acute viral hepatitis was made, and treatment started, including Intravenous fluids, Fresh frozen plasma (FFP) transfusion and injection of Vitamin K.

On hospital days 1 and 2, anorexia nausea did not improve. Still, fortunately, she did not develop any mental changes and no evidence of bleeding. However, liver chemistries and coagulation studies showed the following changes: Alkaline phosphatase (ALP) was 266 U/L, AST 1163 U/L, alanine aminotransferase (ALT) 765 U/L, total bilirubin 19.16 mg/dL, international normalized ratio (INR) 1.9. Viral causes for acute hepatitis were negative, including hepatitis A, B, C, and E. As early treatment with N‐acetylcysteine has been shown to improve survival in those with non‐acetaminophen liver failure, this was also initiated. N‐acetylcysteine was employed as a solution for internal use at a dose of 150 mg/kg (0.75 mL/kg) as the starting dose, followed by a maintenance dose regimen. Abdominal ultrasound revealed a normal, mildly enlarged liver with an edematous gallbladder, as seen in viral infections, without evidence of gallstones.

On day 3, clinical symptoms improved, but liver functions continued to remain deranged. Gastroenterology was also taken on board, and with mutual consensus, keeping in mind other causes of acute hepatitis, an extensive workup was sent, which showed ANA was negative. Liver/kidney microsomal antibody and smooth muscle antibody serology were (1.17 U/mL) normal. Serum alpha 1 antitrypsin was regular. Serum ceruloplasmin was normal.

## 4 CONCLUSION AND RESULTS (OUTCOME AND FOLLOW‐UP)

Over the next week, clinical conditions and the patient's liver enzymes and coagulation studies significantly improved, but total bilirubin remained high. MRCP was done, which showed hepatomegaly and an edematous gallbladder. The pancreatic duct, common bile duct, and intrahepatic ducts were standard. As all workups showed normal results, severe acute respiratory syndrome coronavirus 2 (SARS‐CoV‐2) antibodies were tested, which were significantly high, 9.92. Significant liver enzymes and coagulopathy improvement occurred after N‐acetylcysteine treatment and supportive care. PCR was not conducted due to its high price at the time. Diseases that have been ruled out by viral hepatitis testing, including EBV and CMV, types A, B, C, D, and E, all came back negative. The symptoms of the patient's thalassemia did not intensify over the duration of the illness. The patient was discharged home after 10 days of hospital stay with much clinical improvement.

## 5 DISCUSSION

Hepatic involvement in acute SARS‐CoV‐2 infection has been extensively documented in adults, irrespective of respiratory symptoms.^7^ However, Hepatic involvement in acute SARS‐CoV‐2 infection in the pediatric population is less extensively studied, with most reported cases indicating mild elevation of liver enzymes.^8^ Liver profile abnormalities are observed in 16%–78% of individuals infected with SARS‐CoV‐2.^9^ Instances of severe acute hepatitis or acute liver failure emerging as the primary manifestation of SARS‐CoV‐2 infection, without substantial respiratory or systemic involvement, are exceptionally rare.^10^ Demographic variables should also be considered regarding hepatic involvement during acute SARS‐CoV‐2 infection.^11^

A retrospective study involving PCR‐positive COVID‐19 patients across three New York‐Presbyterian hospitals utilizing alanine aminotransferase (ALT) abnormalities to categorize the level of liver involvement and enhance the understanding of clinical outcomes.^12^ The classification of liver disease severity was based on ALT levels: mild if elevated but less than two times the upper normal limit (UNL), moderate if ALT was between two and five times UNL, and severe if ALT exceeded five times UNL.^12^

Currently, there is no established standard treatment protocol for COVID‐19 Presenting as acute viral hepatitis. Current approaches involve implementing infection control measures, providing symptomatic treatment, and offering supportive care, such as supplemental oxygen and mechanical ventilation when necessary.^13^ The multidisciplinary approach in our case, involving gastroenterology consultation and an extensive workup, was crucial in ruling out alternative etiologies and guiding appropriate management. The use of N‐acetyl cysteine, despite the absence of acetaminophen toxicity, underscores the importance of considering broader applications of therapeutic interventions, especially in the context of severe liver dysfunction.

This underscores the need for further exploration into the unique aspects of pediatric COVID‐19‐related hepatic manifestations, their underlying mechanisms, and potential implications for clinical management.

In summary, this case reveals a rare presentation of severe hepatic involvement in a 4‐year‐old with COVID‐19 and thalassemia minor. The unexpected SARS‐CoV‐2 antibody elevation and unconventional treatment underscore the need for heightened awareness in addressing atypical manifestations, urging further exploration into COVID‐19's nuanced impact on pediatric patients with underlying conditions.

## AUTHOR CONTRIBUTIONS

**Syed Abdan Jamalvi:** Conceptualization; writing – original draft; writing – review and editing. **Sameer Abdul Rauf:** Writing – original draft; writing – review and editing. **Atika Sherali:** Resources; writing – original draft; writing – review and editing. **Syed Khizar Ali:** Writing – original draft; writing – review and editing. **Hussain Haider Shah:** Supervision; writing – original draft; writing – review and editing. **Filza Jamalvi:** Writing – original draft; writing – review and editing. **Fnu Yogeeta:** Writing – original draft; writing – review and editing. **Tirth Dave:** Project administration; resources; supervision; writing – original draft; writing – review and editing.

## FUNDING INFORMATION

None.

## CONFLICT OF INTEREST STATEMENT

None declared.

## ETHICS STATEMENT

Ethical approval was not required for the case report as per the country's guidelines.

## CONSENT

Written informed consent was obtained from the patient for publication and any accompanying images. A copy of the written consent is available for review by the Editor‐in‐Chief of this journal on request. Written informed consent was obtained from the patient's parents/legal guardian for publication and any accompanying images. A copy of the written consent is available for review by the Editor‐in‐Chief of this journal on request.

## Data Availability

The data supporting this article's findings are available from the corresponding author upon reasonable request.
